# Septic arthritis of the hip joint due to *Bacteroides fragilis* in a paraplegic patient

**DOI:** 10.1099/acmi.0.000071

**Published:** 2019-10-21

**Authors:** Anna Shalman, Asaf Acker, Alexander Shalman, Dmitry Frank, Abraham Borer, Leonid Koyfman, Vladimir Kotlovker, Lisa Saidel-Odes, Ohad Gabay, Moti Klein, Evgeni Brotfain

**Affiliations:** ^1^​ Department of Anesthesiology and Critical Care, Soroka Medical Center, Ben-Gurion University of the Negev, Beer Sheva, Israel; ^2^​ Orthopaedic Surgery Department, Soroka Medical Center, Ben-Gurion University of the Negev, Beer Sheva, Israel; ^3^​ Department of Radiology, Barzilai Medical Center, Ben-Gurion University of the Negev, Beer Sheva, Israel; ^4^​ Infection Control Unit, Soroka Medical Center, Ben-Gurion University of the Negev, Beer Sheva, Israel

**Keywords:** *Bacteroides fragilis*, septic arthritis, native joint, critical care

## Abstract

Septic arthritis of native joints is a potentially life-threatening disease. The most frequently isolated pathogens are Gram-positive cocci. *Bacteroides fragilis* is a rare pathogen in joint infections and is usually associated with immunocompromised and debilitated patients. Most cases of *B. fragilis* joint infection are related to skin or local perineal infections or are secondary to *B. fragilis* bacteraemia from another source, for example from the gastrointestinal tract. We present a clinical case of *B. fragilis* septic arthritis involving a native hip joint in a previously healthy paraplegic patient.

## Introduction

Septic arthritis of native joints is a potentially life-threatening pathological state associated with morbidity and mortality [1]. The most frequently isolated pathogens are *Staphylococcus aureus* accounting for more than 50 % of reported cases, streptococci causing almost 25 % of cases, and Gram-negative bacilli isolated in 10–15 % of cases [2, 3]. Anaerobic infections are a rare cause of isolated septic arthritis [1–4]. *Bacteroides fragilis* is an anaerobic, Gram-negative rod-shaped bacterium commonly present in the gastrointestinal tract [4–6]. Joint infection caused by *B. fragilis* is very rare, reported in immunocompromised and debilitated patients with underlying gastrointestinal or skin disease, rheumatoid arthritis, chronic alcoholism, congestive heart failure, Hodgkin lymphoma or sickle cell disease [7, 8]. Most of these cases were attributed to haematogenous spread, chronic skin or local perineal infections [7, 8]. We present a case of septic arthritis of a native hip joint in a previously healthy patient caused by *B. fragilis *.

## Case Report

A 64-year-old male was admitted to the orthopaedic ward with fever. He had a past medical history of smoking, nephrolithiasis and paraplegia following a motor accident 30 years ago. The patient suffered a mid-shaft fracture of his left femur 12 months earlier, without dislocations, treated conservatively with no operation. There were no recent gastrointestinal events, skin diseases or pressure sores.

A computed tomography (CT) scan on admission demonstrated avascular necrosis of the left hip joint with joint destruction and many fluid collections in the soft tissue around the joint. Blood tests showed a white blood cell (WBC) count of 20 210 µl^−^^1^, and a C-reactive protein level of 29.5 mg dl^−^^1^. Synovial fluid tapping revealed a WBC count of 80 000 µl^−^^1^. Synovial fluid cultures and blood cultures were sent for analysis.

Septic arthritis of the left hip joint was diagnosed and the patient was treated with antibiotics including metronidazole and underwent surgical drainages of the hip. After initial surgical drainage, the patient developed multiple organ failure including cardiovascular instability, decreased urine output and acute respiratory failure due to septic shock and was admitted to our general intensive care unit (GICU). During the next 4 days in the intensive care unit, the patient continued to be septic with elevated temperatures (39.2 °C) and continuous leukocytosis (20 000 µl^−^^1^). During this period, intensive supportive management included the administration of vasopressors and broad-spectrum antibiotics.

**Fig. 1.**
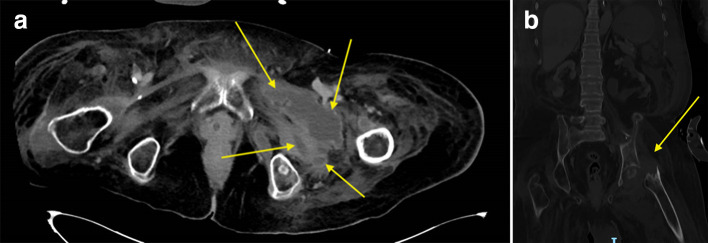
(a) CT axial image at the level of the hips demonstrating multiple inoculated abscesses around the left hip joint area (yellow arrows). (b) CT image in the coronal plane showing complete destruction of the left hip joint (yellow arrow), visualized under Bone window.

On a detailed workup of sepsis, multiple bloods, sputum, wound and stool cultures were obtained. Diagnosis of septic arthritis was confirmed by a positive synovial fluid for *B. fragilis *. A repeated CT scan of the left hip, pelvis and lower abdomen with contrast media demonstrated multiple, inoculated abscesses around the left hip joint area spreading to the left flank and anterior abdominal wall (Fig. 1). No evidence of colitis or other potential intra-abdominal source of septic arthritis was encountered in the CT images.

During the next week, multiple abscesses were percutaneously drained under CT guidance. The patient's status improved and a weaning process from mechanical ventilation was initiated. However, 2 weeks later, repeated CT imaging showed a recurrence of multiple intra-articular abscesses. The patient was transferred to the operating room and a left extended hemipelvectomy was performed. A culture of pus obtained at surgery around the iliopsoas muscle was also positive for *B. fragilis *. All blood cultures were negative. *B. fragilis* was identified using thioglycollate broth, and the organism was cultured on Brucella agar. Further characterization was performed with VITEK MS (bioMérieux). We did not perform sensitivity testing for anaerobic bacteria, but a lactamase test was used.

The patient continued antibacterial therapy with intravenous metronidazole 500 mg QID during his entire GICU stay. The patient was discharged from the unit 8 weeks after admission with a WBC count of 4800 µl^−^^1^ and a C-reactive protein level of 1.5 mg dl^−^^1^.

## Discussion

*B. fragilis* is non-spore-forming Gram-negative bacillus that is a part of the human normal flora [3, 5, 6, 9]. *Bacteroides *, the predominant bacteria found in the human intestine, are usually isolated from the colon, although infections caused by or associated with them can include virtually any organ [3, 5, 9, 10]. Multiple virulence factors have been implicated in the pathogenesis of this organism: the capsular polysaccharide (which inhibits opsonophagocytosis and promotes abscess formation), pili and fimbriae (promote adherence), and production of a number of different enzymes (hyaluronidase, haemolysin, peroxidase, collagenase, protease, heparinase and neuraminidase) [3, 9]. *B. fragilis* is the most common anaerobic bacterium isolated from blood cultures, although the frequency of positive cultures continues to decrease overall. In virtually all cases, isolation of a member of the *B. fragilis* group in blood cultures indicates an underlying infection, and is associated with 60 % mortality if left untreated. The source of bacteraemia is most commonly intra-abdominal, the female genital tract or soft tissue [8, 9]. Other relevant but less frequent conditions are respiratory tract infections, endocarditis, pericarditis, meningitis and osteoarticular infections [6–9]. Septic arthritis of native joints due to *B. fragilis* is extremely rare. Most of these cases of *B. fragilis* septic arthritis have been attributed to haematogenous spread from an infected focus, mostly intra-abdominal [9]. Septic arthritis caused by *B. fragilis* usually affects older patients in their 5th and 6th decade, of whom over 90 % have concomitant multiple risk factors or underlying diseases such as ischaemic cardiomyopathy, diabetes mellitus, neoplasia, blood dyspraxia, corticosteroid therapy, or immunosuppressant or joint disease such as rheumatoid arthritis, and haematic dissemination from an infected site. Sites of seeding have included colon tumours, intestinal anastomosis, appendicular abscess, gangrenous appendicitis and pilonidal cyst resection [8, 9]. Septic arthritis due to *B. fragilis* may be followed by joint destruction and usually affects the knee, elbow, ankle or hip [8, 9].

In the present case, we describe a patient with primary septic hip arthritis caused by *B. fragilis *. He had no underlying acute infectious process, chronic disease or affected joints. Moreover, in our patient, *B. fragilis* arthritis developed in a native joint.

Despite no evidence of potential risk factors for developing septic arthritis, the patient was paraplegic after spinal cord injury 10 years previously. We found no previously published evidence of increased incidence of septic arthritis due to *B. fragilis* in paraplegic patients. However, there is a strong correlation between spinal cord injury (SCI) (and paraplegic state) and extensive bone loss in hip joints associated with increased levels of osteoclasts and their activity, which facilitate bone absorption [10, 11].

As is the case with most people with osteoporosis, patients with SCI are asymptomatic until a fracture occurs. The absence of pain and a history of minor or no trauma will make the diagnosis of fracture somewhat difficult. In the asensate limb, a fracture may be suspected on the basis of the presence of localized swelling, deformity, autonomic symptoms, etc., but the diagnosis is confirmed by radiological studies [10]. Whylie *et al*. [12] reported that about 70 % of patients with cervical spine injury and up to 20 % with lumbar spine injury developed moderate joint degeneration.

Despite the fact that fractures in a paralysed limb are relatively rare [13], such asymptomatic, subclinical fracture of the hip joint might be a potential risk factor for the development of septic arthritis.

We treated our patient according to well-described guidelines for septic arthritis [14, 15] and in accordance with synovial cultures positive for *B. fragilis *. However, intravenous antibacterial therapy together with CT-guided drainage of multiple abscesses was not successful in the present case and, finally, our patient underwent radical hemipelvectomy. Two months after hospitalization the patient was doing well, and antibiotic treatment was stopped with no further signs of infection.

### Conclusion

Septic arthritis due to *B. fragilis* infection is a life-threatening disease that might be complicated by potential multiple organ failure. We have presented an interesting case of isolated *B. fragilis* infection affecting a native hip joint in a paraplegic patient. In such cases, early antibacterial therapy combined with radiological drainages and surgical intervention can lead to significant improvement in the patient's outcome.
